# Calibrated Uncertainty Estimation for Soil Organic Carbon from Raman Spectra

**DOI:** 10.1021/acs.analchem.5c04616

**Published:** 2025-12-11

**Authors:** Jeffrey K. Wiens, Natalia Solomatova, Sadegh Shokatian

**Affiliations:** Miraterra Technologies Corporation, 199 W Sixth Avenue, British Columbia, Vancouver V5Y 1K3, Canada

## Abstract

Machine learning (ML) is a powerful tool for inferring chemometric properties from Raman spectra, expanding the information extractable from high-dimensional spectral data. A growing application is the estimation of soil organic carbon (SOC), where ML models relate overlapping Raman and fluorescence features to chemical composition. However, these models typically lack calibrated, prediction-level uncertainty estimates that limit their utility in decision-critical contexts. We present a framework for quantifying predictive uncertainty in SOC estimation from Raman spectra using Shifted Excitation Raman Difference Spectroscopy (SERDS). The approach employs conformal prediction (CP) to generate statistically valid prediction intervals using a held-out calibration data set and is compatible with a variety of uncertainty quantification (UQ) methods. To our knowledge, this is the first unified framework that integrates conformal calibration with multiple UQ strategies for Raman-based SOC estimation, addressing both aleatoric (irreducible) and epistemic (reducible) sources of uncertainty in a field-relevant setting. We assess the framework across several regression models, including Deep Ensembles, Bayesian neural networks, Monte Carlo Dropout, quantile regression, and heteroscedastic Gaussian models. All methods, when conformalized, produced well-calibrated uncertainty estimates with narrow prediction intervals, achieving reliable empirical coverage across confidence levels. Ablation studies revealed that many UQ techniques were poorly calibrated without conformalization. Our findings indicate that uncertainty in this task is predominantly aleatoric in nature, suggesting that improvements in predictive performance will depend more on improving spectral quality and preprocessing than on model complexity. This framework provides a practical, generalizable solution for generating trustworthy, calibrated, sample-specific uncertainty estimates in Raman-based chemometric analyses.

## Graphical abstract

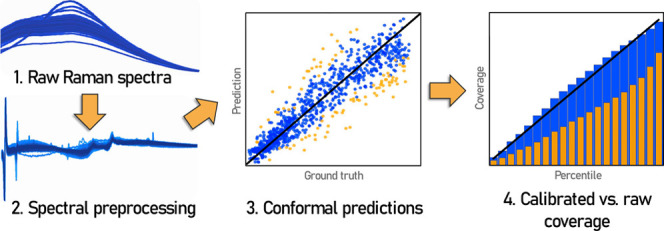

## Machine Learning and Uncertainty in Soil Spectroscopy

Machine learning (ML) plays an increasingly central role in modern chemometrics, enabling multivariate, nonlinear relationships to be inferred directly from spectral data. Among optical techniques, Raman spectroscopy stands out as a label-free, nondestructive method that captures molecular information by probing vibrational modes through shifts in the energy of the scattered light. When paired with ML, Raman spectra can be used to model a wide range of chemical and physical properties, extending the analytical reach of spectroscopy across diverse application domains. One such application is the estimation of soil organic carbon (SOC), a key indicator of soil health and fertility.^1–5^

A frequent shortcoming of ML in analytical settings is the lack of calibrated, prediction-specific uncertainty estimates, which are essential for precision measurements and confident interpretations. In spectroscopic analysis, uncertainty is typically heteroscedastic, meaning its magnitude depends on the input, and arises from both aleatoric (irreducible) and epistemic (reducible) sources.^6^ While global error metrics offer a coarse view of model performance, they obscure variation across data strata, often leaving practitioners with an incomplete understanding of model reliability. This challenge is exacerbated by the black-box nature of many modern ML models, which offer limited interpretability and little transparency into prediction confidence.

These challenges are especially pronounced in SOC estimation from the Raman spectra of soils. Soil is highly heterogeneous, containing a mixture of organic matter, microbial and plant-derived residues, and minerals. Many of these components, including the organic matter, microbial metabolites, and decomposing biomass, exhibit fluorescence under near-infrared excitation. As a result, fluorescence is consistently present and overlays the Raman signal with significantly greater intensity, presenting numerous measurement challenges and sources of uncertainty.^7^ Some of these issues can be mitigated by Shifted Excitation Raman Difference Spectroscopy (SERDS)^8–10^ and advanced signal processing techniques such as common-mode rejection.^5^ Nevertheless, even after fluorescence reduction, the relationship between spectral features and SOC remains difficult to model. This is due to the diversity of mineral-organic compositions and the corresponding albedo effects, which can produce similar spectral responses for soils with different SOC levels. As a result, the mapping from spectrum to SOC can be multimodal, with multiple plausible SOC estimates corresponding to a single spectral measurement.

In this work, we introduce a general framework for calibrated uncertainty quantification in spectroscopic regression models, with a focus on SOC estimation from SERDS-acquired Raman spectra. We apply conformal prediction (CP), a distribution-free method that calibrates prediction intervals using a held-out data set to guarantee valid coverage under minimal assumptions.^11–13^ This enables a model-agnostic uncertainty calibration without requiring changes to the underlying predictive model. The framework is validated across a range of representative ML-base UQ techniques, spanning probabilistic^14–16^ and deterministic^17–19^ approaches. Our results provide insight into the dominant sources of uncertainty in soil spectroscopy and highlight pathways for improving the reliability and interpretability of spectroscopic SOC estimation models. These findings are applicable across different spectroscopic approaches due to shared sample-level characteristics, such as overlapping signals and reflectance-driven effects, as well as the method’s independence from model architecture and minimal assumptions about data distribution.

## Materials and Methods

### Sample Preparation and Data Collection

The data set comprises 901 soil samples collected from 175 fields across North America. This is the same data set used in Zarei et al.,^5^ which can be referred to for additional methodological details. Samples were obtained from both Western Canada and the Midwestern United States, capturing a variety of soil types, climates, and agricultural practices. Each sample was processed according to industry protocols, including air drying, gentle grinding, and sieving to 2 mm. Ground-truth SOC values were measured via dry combustion and ranged from 0.28% to 6.95%.

Raman spectral data were collected using Miraterra’s soil digitizer, which is equipped with dual near-infrared lasers. To account for within-sample soil heterogeneity, measurements were collected over a 10 × 10 spatial grid. At each grid position, alternating Raman spectra were acquired using two closely gapped lasers with a 200 ms integration time, 25 mW power, and 15 replicates. This collection procedure resulted in a total of 3000 measurements per sample.

### Spectral Preprocessing Techniques

Following Zarei et al.,^5^ we applied Multiplicative Scattering Correction (MSC)^20^ across the spectra of each replicate set. MSC is a preprocessing technique that reduces variability caused by additive and multiplicative scattering effects, often arising from differences in particle size, surface roughness, or optical path length. It does so by aligning each spectrum to a reference using linear correction factors, thereby minimizing baseline shifts and scaling artifacts. After MSC, the replicates were averaged and normalized by the integration time yielding a single spectrum per replicate set. Next, we applied a variation of SERDS^8,9^ to the two processed laser measurements at each sample grid position. This is done by first applying common-mode rejection,^5^ a baseline correction technique that uses wavelet decomposition and regression alignment to isolate and remove shared background artifacts between the two spectra, thereby enhancing sample-specific spectral differences. Next, the intensity of the two spectra was subtracted from each other to remove the fluorescence background. The resulting difference spectra (a derivative-like signal) were averaged across sample grid positions and smoothed using a Savitzky–Golay^21^ smoothing filter with a window size of 11 points and polynomial order of 2. Finally, all spectra were resampled onto a uniform wavenumber grid ranging from 350 to 2000 cm^–1^ with a 1 cm^–1^ step size, ensuring consistent feature representation across all samples. This resulted in 1650-channel spectral inputs for the model.

### Conformal Prediction Framework

Our uncertainty quantification methodology is based on split-conformal prediction^11,12^ using a variant of conformalized quantile regression.^13^ Split-conformal prediction calibrates a model’s uncertainty estimates using a held-out calibration set to guarantee coverage while conformalized quantile regression applies this to quantile-based models by adjusting intervals based on residuals from the calibration data. This approach provides statistically valid prediction intervals that do not rely on assumptions about the underlying data distribution or model architecture.

The framework employs a black-box model f̂ that produces point predictions and uncertainty estimates ŷ,σ̂=f̂(x;θ) where *ŷ* is the predicted SOC value, σ̂ is the estimated standard deviation of the SOC prediction, and θ are model parameters. This formulation is compatible with a range of uncertainty estimation techniques described below. Using these outputs, we first construct uncalibrated prediction intervals Ĉ(x)=[ŷ−zσ̂,ŷ+zσ̂] , where *z* ≈ 1.64 corresponds to 90% coverage assuming a normal distribution of errors. By constructing prediction intervals in this manner, we are assuming that error residuals follow a Gaussian distribution.

To calibrate these intervals, we use a held-out calibration set (*X*_cal_, *Y*_cal_) consisting of *n* i.i.d. samples. For each calibration example, we compute a nonconformity score (1)

1 $$s\left(x_{i},y_{i}\right)=\max\left\{y_{i}-\left({ŷ}_{i}+z{\mathrm{\sigma ̂}}_{i}\right),\left({ŷ}_{i}-z{\mathrm{\sigma ̂}}_{i}\right)-y_{i}\right\}$$

where *x*_*i*_ and *y*_*i*_ are the spectral input and ground-truth SOC value for the *i*th calibration example, respectively, and ${ŷ}_{i},{\mathrm{\sigma ̂}}_{i}=\mathrm{f̂}\left(x_{i};\theta \right)$ . The nonconformity score represents the distance between the true value and the nearest interval boundary, where the score is negative when the true value falls within the predicted interval and positive when outside. Here, a smaller absolute-valued nonconformity score indicates better model calibration. The final, calibrated prediction intervals are adjusted based on the empirical distribution of these nonconformity scores. We compute a quantile *q̂*, defined by (2), from the distribution of nonconformity scores, which serves as an empirical adjustment factor to calibrate the prediction intervals

2 $$\mathrm{q̂}=Quantile\left({\left\{s\left(x_{i},y_{i}\right)\right\}}_{i=1}^{n},\frac{\left[\left(n+1\right)\left(1-\alpha \right)\right]}{n}\right)$$

where α = 0.1 for 90% coverage. The value *q̂* represents the (1 – α) upper quantile of the nonconformity scores and ensures that at least 1 – α of the calibration examples fall within the adjusted interval. This adjustment factor *q̂* is then used to expand the original prediction intervals using (3)

3 C(x)=[ŷ−zσ̂−q,ŷ+zσ̂+q]

The conformal calibration guarantees that P(y∈C(x))≥1−α for any new test point (*x*,*y*) drawn from the same distribution as the calibration data. This property holds regardless of the accuracy or degree of miscalibration of the original model *f̂*. Furthermore, the calibration can be incorporated directly into the model’s predicted standard deviation by setting $\sigma =\mathrm{\sigma ̂}+\frac{\mathrm{q̂}}{z}$ . The resulting calibrated standard deviation σ can then be used to construct prediction intervals at any desired confidence level, with the guarantee that the 90th percentile interval will have at minimum the specified coverage probability. If the prediction error follows a Gaussian residual, all constructed prediction intervals should be well-calibrated.

### Uncertainty Quantification Methods

Although conformal prediction is valid for any heuristic notion of uncertainty, the quality of the uncertainty estimates can vary greatly depending on the choice of uncertainty heuristic. In this work, we consider two types of UQ methods: deterministic methods that directly output uncertainty estimates alongside predictions, and probabilistic methods that estimate uncertainty by sampling multiple predictions from stochastic models. Deterministic methods are well-suited for quantifying aleatoric uncertainty, which is the uncertainty associated with the inherent noise or ambiguity of the input data. Probabilistic methods, by contrast, are designed to capture epistemic uncertainty, which is the uncertainty associated with the model’s parameters due to limited data. For deterministic methods, we consider quantile regression^13,18^ and a heteroscedastic Gaussian model,^17,19^ both of which explicitly model prediction variability as a function of the input. For probabilistic methods, we consider Monte Carlo (MC) Dropout,^15^ Bayes by Backprop,^14^ and Deep Ensembles,^16^ all of which produce multiple predictions for a given input by introducing randomness during inference. A detailed description of the methods can be found in the Supporting Information. Note that for the case of quantile regression, we apply a nonstandard variation of the method that enables the standard deviation to be directly computed.

### Convolutional Neural Network Architecture

In this work, we consider a standard 1D convolutional neural network (CNN) architecture to process the input Raman spectra, each consisting of 1650 channels. The model consists of multiple convolutional blocks followed by several dense (fully connected) layers for higher-level representation and regression. Each convolutional block consists of a 1D convolutional layer, a ReLU (rectified linear unit) activation function, batch normalization to stabilize training, and max-pooling to reduce dimensionality. After the final convolutional block, the resulting feature maps are flattened into a single vector and passed through a sequence of dense blocks, each consisting of a fully connected layer with a ReLU activation function, and dropout regularizer to prevent overfitting. The final dense layer outputs one or two values depending on the UQ method, which are the predicted SOC value and the estimated standard deviation. The estimated standard deviation is only used for the deterministic UQ methods. Probabilistic UQ methods rely on repeated sampling to estimate uncertainty and therefore do not require an explicit variance output.

For each UQ method, we tuned the architecture so that the model’s accuracy was comparable across all methods. When possible, model and preprocessing hyperparameters were initialized to values used by Zarei et al.^5^ No exhaustive hyperparameter searches were performed. Hyperparameters were hand-tuned with the explicit goal of replicating Zarei et al.^5^ performance results. The exact architecture is provided in the Supporting Information.

### Model Training Procedure and Validation

We employ 10-fold cross-validation to evaluate the performance of each uncertainty estimation method. For each cross-validation fold, we train ten different models, each with a different training and calibration set. The calibration set is created by randomly selecting 100 samples from the training-folds, which are used solely for conformal calibration and are not seen during model training. The remaining samples are used to train the model. We also evaluated a field-based variant of cross-validation to avoid leaking information from correlated samples between the training and validation set; however, this approach had no significant effect on the results and was therefore not included in the final analysis.

To provide a representative assessment of uncertainty estimates, we evaluate the coverage of each calibrated model on the held-out validation data and select the model with the median coverage error (defined as the absolute difference between the empirical coverage and the nominal confidence level) for the 90th-percentile. By selecting the model with the median coverage error, we ensure that the reported results are representative of the typical performance of the uncertainty estimation method. The predictions are combined across all folds (one prediction for each sample in the entire data set) to produce our aggregated results.

## Results and Discussion

We first evaluate the predictive accuracy of each UQ method. As shown in Table 1, all models perform similarly by design and are consistent with previous results reported in Zarei et al.^5^ Figure 1 shows the predicted vs ground-truth SOC values for two representative models, with 90% confidence intervals indicated.

**Table 1:** Prediction Performance Metrics Across all UQ Methods

Model	*R* ^2^	MAE	MAPE (%)	RMSE
Heteroscedastic Gaussian	0.842	0.569	24.02	0.762
Quantile Regression	0.857	0.541	23.37	0.725
Bayes By Backprop	0.865	0.516	21.18	0.704
MC Dropout	0.842	0.559	21.95	0.763
Deep Ensembles	0.866	0.528	22.31	0.702

**Figure 1:**
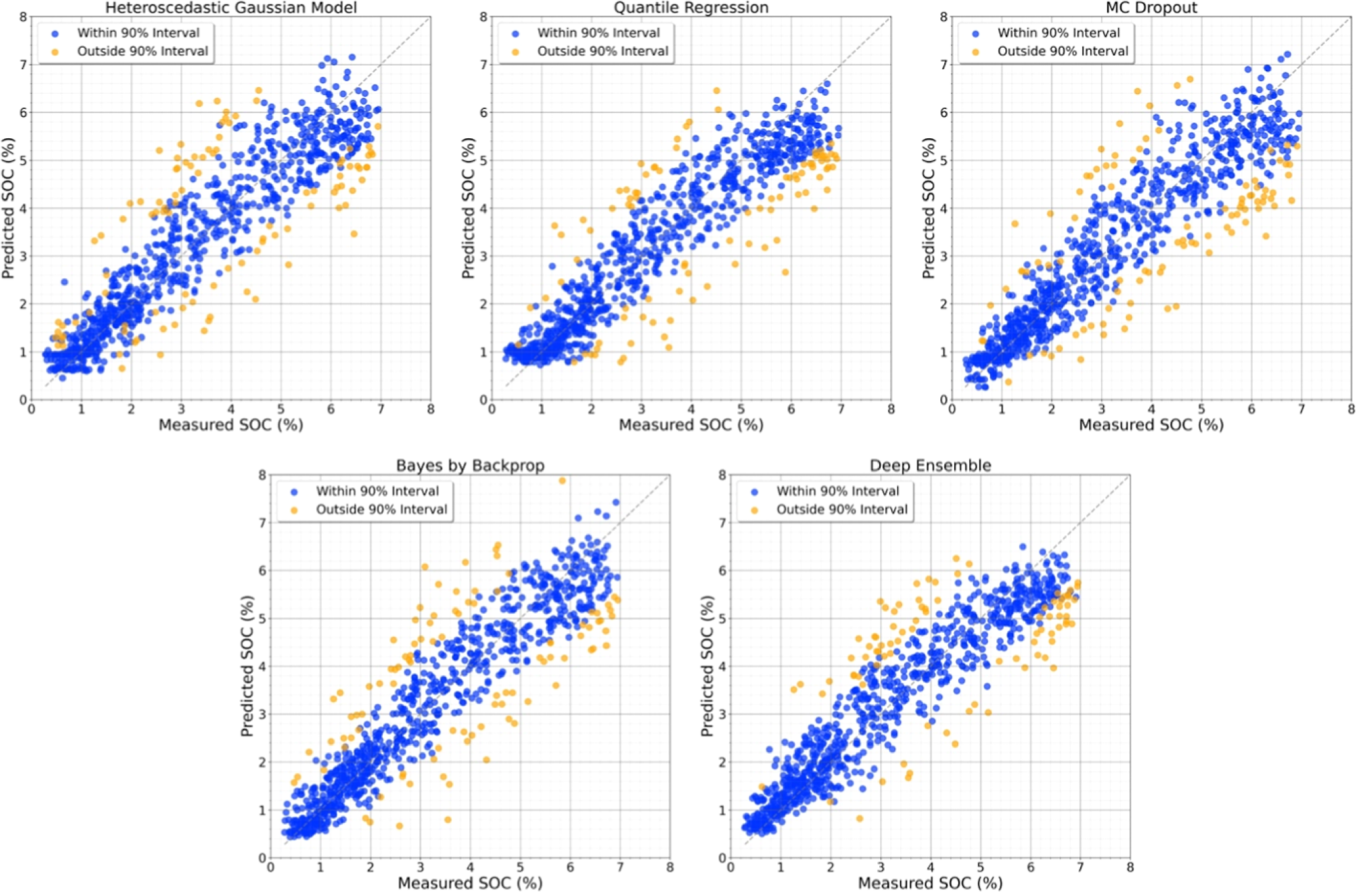
Prediction vs ground truth SOC values for two representative models. Each point corresponds to a soil sample, where blue indicates samples within the 90% confidence interval, and orange indicates those outside.

We next evaluate the quality of the uncertainty estimates following conformalization, as reported in Table 2. All models exhibit strong calibration, with empirical coverage remaining within 2% of the target 90% confidence level. In addition to coverage, we assess three properties of the uncertainty estimates: sharpness, consistency, and informativeness of our uncertainty estimates. We define sharpness as the median width (i.e., standard deviation) of the predictive interval; narrower widths correspond to more precise predictions. Consistency is defined as the variance of the predicted interval widths; lower variance indicates more stable model behavior. Informativeness is defined as the correlation between predicted uncertainty and absolute prediction error; higher correlation indicates greater risk sensitivity.

**Table 2:** Uncertainty Metrics after Conformalization Across all UQ Methods

model	coverage (90%)	Median[σ̂]	Var[σ̂]	Corr[|error|,σ̂]
Heteroscedastic Gaussian	0.887	0.685	0.0472	0.409
Quantile Regression	0.898	0.675	0.0255	0.302
Bayes By Backprop	0.892	0.658	0.0231	0.252
MC Dropout	0.895	0.704	0.0568	0.329
Deep Ensembles	0.905	0.671	0.0239	0.221

Sharpness values are similar across methods and generally align with each model’s mean absolute error (MAE) and mean absolute percentage error (MAPE), indicating a tight coupling between predictive error and uncertainty width. For instance, Bayes by Backprop yielded both the sharpest uncertainty estimates and lowest MAE, followed by Deep Ensembles. Given this correlation, observed differences in sharpness likely result from the model’s underlying predictive performance and could potentially be reduced through hyperparameter tuning.

The informativeness of the uncertainty estimates are more pronounced in the heteroscedastic Gaussian and MC Dropout models. Both had the highest variance in predicted standard deviation and the strongest Pearson correlation with absolute error, suggesting improved risk sensitivity. Between the two, the heteroscedastic Gaussian model was more consistent and informative overall. The correlation with absolute error is visualized in Figure 2.

**Figure 2:**
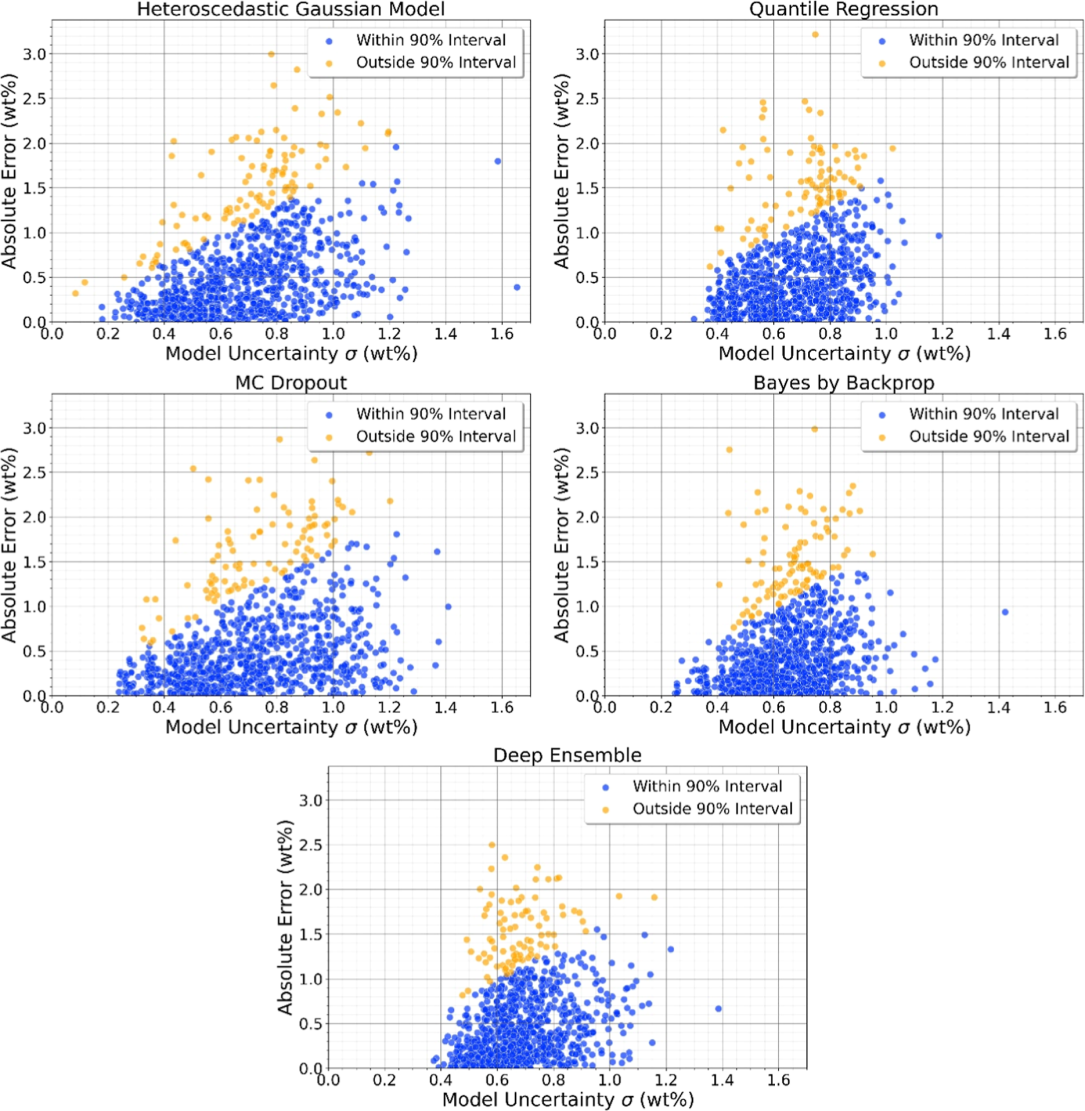
Absolute error vs predicted standard deviation. Each point represents a soil sample, where blue indicates samples within the 90% confidence interval, and orange indicates those outside.

In Table 3a, we assess the empirical coverage of the predicted uncertainty estimates across a range of confidence levels. Overall calibration quality is summarized using the root mean squared error (RMSE) between the empirical and nominal coverage from 5% to 95% in 5% increments, with an additional evaluation at 99%. Although conformalization was applied only at the 90th percentile, all methods achieve near-ideal coverage across the full range of confidence levels.

**Table 3:** Coverage at Different Confidence Levels for Models with and Without Conformalization^a^

	RMSE	10%	20%	50%	80%	90%	95%	99%
(a) model (after CP)								
Heteroscedastic Gaussian	0.010	0.088	0.196	0.499	0.789	0.887	0.935	0.978
Quantile Regression	0.021	0.102	0.221	0.535	0.814	0.898	0.940	0.978
Bayes By Backprop	0.037	0.123	0.234	0.550	0.799	0.892	0.931	0.976
MC Dropout	0.032	0.098	0.212	0.552	0.819	0.895	0.932	0.980
Deep Ensembles	0.045	0.115	0.246	0.574	0.814	0.905	0.942	0.973
(b) model (before CP)								
Heteroscedastic Gaussian	0.011	0.093	0.205	0.503	0.800	0.886	0.943	0.978
Quantile Regression	0.031	0.090	0.194	0.471	0.750	0.861	0.913	0.962
Bayes By Backprop	0.226	0.060	0.108	0.281	0.483	0.579	0.645	0.759
MC Dropout	0.213	0.050	0.107	0.299	0.499	0.598	0.674	0.769
Deep Ensembles	0.136	0.065	0.131	0.376	0.607	0.713	0.779	0.875
(c) model (before vs after CP)								
Heteroscedastic Gaussian	–0.000	–0.006	–0.009	–0.003	–0.011	0.001	–0.009	0.000
Quantile Regression	–0.011	0.012	0.027	0.064	0.063	0.037	0.027	0.016
Bayes By Backprop	–0.189	0.063	0.127	0.270	0.316	0.313	0.286	0.216
MC Dropout	–0.180	0.048	0.105	0.253	0.320	0.296	0.259	0.211
Deep Ensembles	–0.090	0.050	0.115	0.198	0.206	0.192	0.163	0.099

a The delta values, defined as the difference between the post-conformal and pre-conformal results, are included for direct comparison.

To assess the impact of conformalization, we report the empirical coverage without conformalization, as shown in Table 3b. Coverages are illustrated in Figure 3. Several UQ methods, particularly the probabilistic ones, exhibit substantial under-coverage and are poorly calibrated in the absence of conformalization. In contrast, deterministic methods such as quantile regression and the heteroscedastic Gaussian model are well-calibrated across all confidence levels, with conformalization offering only slight improvement near the 90th percentile.

**Figure 3:**
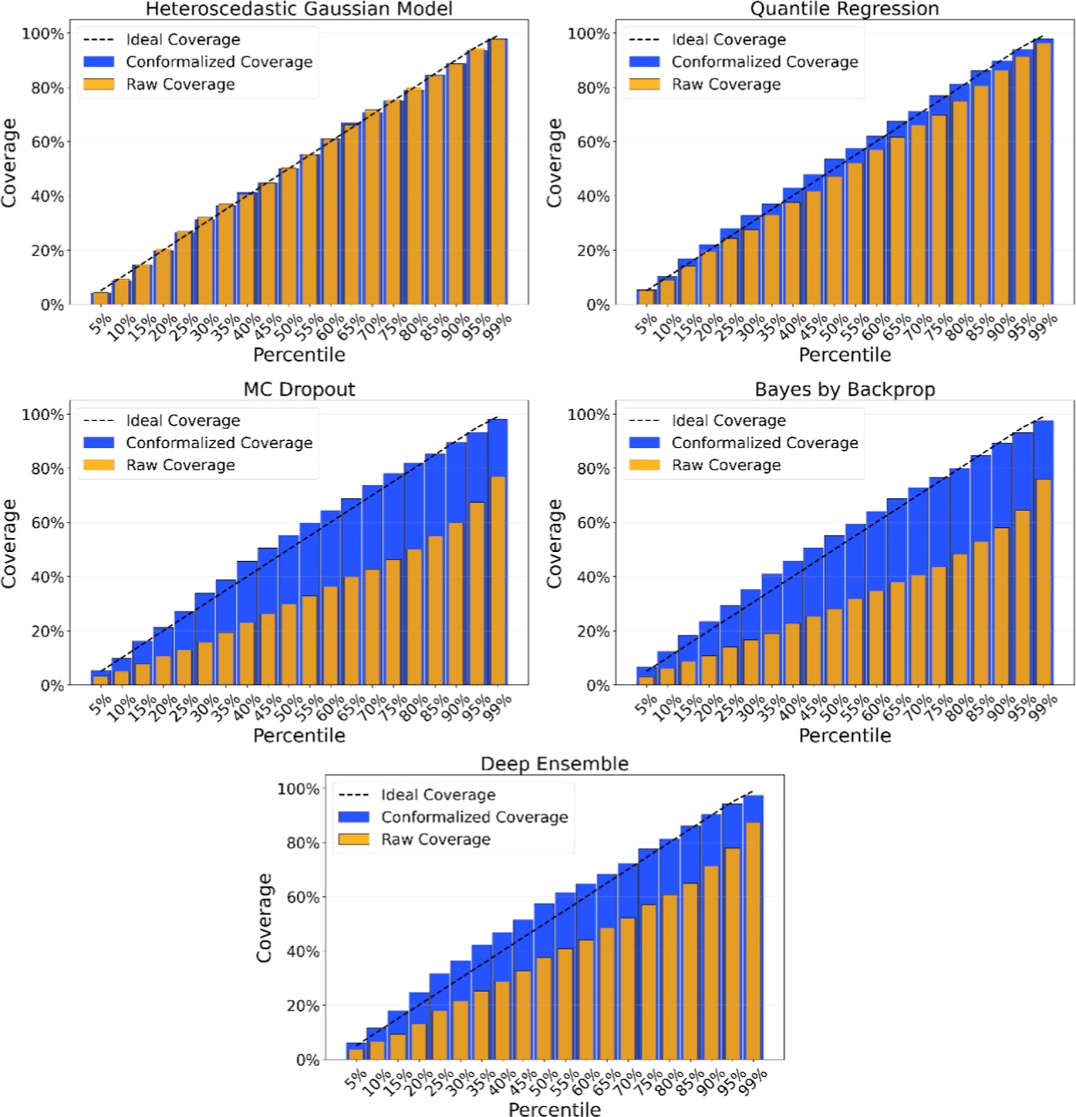
Comparison of coverage with and without conformal calibration across different confidence levels. The diagonal line indicates perfect calibration, where the achieved coverage exactly matches the expected coverage. Deviation from this line reflects underconfidence or overconfidence in the predicted uncertainty intervals.

To interpret these results, we follow the work of Hüllermeier and Waegeman.^6^ We define epistemic uncertainty as the reducible portion of total uncertainty arising from limited knowledge or data, and aleatoric uncertainty as the irreducible portion arising from inherent variability in the data-generating process. Training a ML model involves selecting a single hypothesis from a larger set of hypotheses that minimizes the total risk (i.e., expected loss). Since any learnt hypothesis is only an approximation (e.g., limited training data), multiple plausible hypotheses may exist with comparable empirical risk. Uncertainty in the correct hypothesis gives rise to epistemic uncertainty, whereas aleatoric uncertainty is embedded in all hypotheses since it originates from the data itself and cannot be eliminated through additional training.

Sampling-based methods naturally capture multiple hypotheses. For example, each model in a Deep Ensemble converges to a different local minimum in the loss landscape due to random initialization and stochastic training, yielding a diverse set of plausible hypotheses. Consequently, the variation among ensemble predictions primarily quantifies epistemic uncertainty. Aleatoric uncertainty, by contrast, shapes the loss landscape but does not directly influence the point predictions of individual models. Nevertheless, it can still have a secondary effect on predictive variation, as model hypotheses may respond differently to ambiguities in the data. Still, this incidental capture of aleatoric uncertainty is usually incomplete and poorly calibrated.

Bayesian methods such as Bayes by Backprop and MC Dropout follow a similar epistemic interpretation. By treating model weights as random variables, each sample from the posterior (or variational approximation thereof) yields a distinct hypothesis, and the spread in predictions reflects epistemic uncertainty. While Deep Ensembles may yield more diverse hypotheses due to fewer parametric constraints, the underlying interpretation across these Bayesian approaches remains consistent.

In contrast, models such as the heteroscedastic Gaussian and quantile regression frameworks are explicitly designed to capture aleatoric uncertainty. These models posit a single deterministic hypothesis and instead incorporate uncertainty through additional output heads. These uncertainty estimates are directly optimized during training using tailored loss functions (e.g., negative log-likelihood or pinball loss), and are calibrated against observed prediction errors. As a result, they are well-suited to capturing irreducible, data-driven uncertainty but do not account for epistemic uncertainty.

From this standpoint, we conclude that the dominant uncertainty in this task is primarily aleatoric. The deterministic methods, which are linked to aleatoric uncertainty, are well-calibrated without conformalization. In contrast, the probabilistic methods, which are linked to epistemic uncertainty, are poorly calibrated without conformalization. Furthermore, we observe distinctions between probabilistic methods. The RMSE coverage error for Deep Ensembles is almost half of Bayes by Backprop and MC Dropout. This suggests that Deep Ensembles are implicitly capturing aleatoric uncertainty better than the other probabilistic methods.

## Conclusions

This work presents one of the first systematic evaluations of calibrated uncertainty estimation in Raman-based soil spectroscopy, using methods applicable to both aleatoric and epistemic uncertainty. We introduce a conformal prediction framework for uncertainty quantification within spectroscopic regression models and validate it through the task of estimating soil organic carbon from SERDS-acquired Raman spectra. Using deep learning UQ methods, we generate statistically valid prediction intervals across a diverse set of soil samples. All tested methods achieve the desired coverage levels with comparable sharpness and informativeness. Among them, Bayes by Backprop and Deep Ensemble displayed the best prediction accuracy and sharpest uncertainty estimates, while the heteroscedastic Gaussian and MC Dropout models provided the most informative uncertainty estimates. Our results highlight the importance of selecting UQ strategies that not only produce sharp intervals but also reflect risk sensitivity in practical applications.

Importantly, our analysis shows that uncertainty in SOC prediction is predominantly aleatoric, reflecting inherent ambiguity in the spectral data and measurement process. Probabilistic UQ methods such as Bayes by Backprop and MC Dropout exhibit substantial under-coverage without conformalization (by 22 to 50%), whereas deterministic methods do not. This highlights that our framework not only corrects such deficiencies but also offers diagnostic insight into the nature of the uncertainty. We propose the aleatoric nature of uncertainty may generalize to other Raman-based applications, since spectral signals often contain limited predictive information or hidden by noise. In many real-world chemometric problems, uncertainty is likely driven more by limited information or measurement fidelity than by model underfitting or the need to capture multimodal relationships.

While some gains may be achieved through model refinement, the primary opportunities for improvement appear to be data-driven. This includes better sample selection and preparation, instrument calibration, improved ground-truth labeling, and advanced preprocessing techniques. Such directions align with broader efforts toward data-centric AI.^22^ Another avenue is to integrate signal processing steps into the model architecture, allowing neural networks to learn more efficient representations and reduce information loss, an approach consistent with the information bottleneck principle.^23^ Integrating signal processing steps into the model architecture provides a practical direction for future studies focused on improving both robustness and interpretability in spectroscopic modeling. Other UQ methods, such as evidential regression,^24^ also warrant considerations within the context of conformal predictions. In this work, we considered a broad, yet incomplete, set of UQ methods. Additional UQ methods could be considered in future work.

The calibration framework proposed in this study is broadly applicable to other spectroscopic techniques, including near-infrared (NIR), mid-infrared (MIR), and laser-induced breakdown spectroscopy (LIBS), which face similar challenges related to signal overlap, confounding spectral features and sample heterogeneity.^25–27^ While this work focused on soil organic carbon as a representative use case, the framework is suitable for a wide range of regression problems in spectroscopic analysis where uncertainty quantification is necessary. Its model-agnostic design and minimal distributional assumptions allow integration with diverse predictive models, supporting use in laboratory settings, field-deployable instruments, and regulatory workflows that require calibrated, sample-specific uncertainty for reliable decision making.

## Supplementary Material



## Data Availability

The raw data and associated codes are subject to corporate intellectual property restrictions and are not publicly available at this time. Access to training data may be granted upon reasonable request and in accordance with applicable IP clearance procedures.

## Notes

Detailed description of UQ methods considered in this work, Detailed model architecture and training procedure, Analysis on conditional coverage (PDF)
