# Supplementary material for: Calibrated Uncertainty Estimation for Soil Organic Carbon from Raman Spectra
Source: Anal Chem. 2025 Dec 11;98(1):309–16. doi: 10.1021/acs.analchem.5c04616 (PMC12809652; doi:10.1021/acs.analchem.5c04616)
Supplement: Supplementary file 1 [file ac5c04616_si_001.pdf]

Supporting Information: Calibrated Uncertainty Estimation for Soil Organic Carbon from Raman Spectra

Jeffrey K. Wiens,* Natalia Solomatova, and Sadegh Shokatian

Miraterra Technologies Corporation, 199 W 6th Ave, Vancouver, British Columbia, V5Y 1K3, Canada

E-mail: jeffrey.k.wiens@gmail.com

Contents

UQ Methods **S-3**

CNN Architecture **S-7**

Conditional Coverage **S-9**

UQ Methods

In this section, we provide a detailed exposition of the UQ methods considered in this work. Fundamentally, we consider two distinct approaches to uncertainty estimation: deterministic and probabilistic.

Deterministic methods explicitly produce both the SOC estimate and its associated uncertainty, expressed as $\hat{y}, \hat{\sigma} = \hat{g}_d(x)$. These methods include quantile regression and a heteroscedastic Gaussian model, where the model is specifically trained to predict not only the expected SOC value but also a measure of its uncertainty.

In contrast, probabilistic methods are stochastic, in which we draw multiple predictions by introducing randomness during inference. In this case, we define a stochastic model $g_p(x)$ that produces varying SOC estimates when called with the same input x. By drawing multiple samples $\{\hat{y}_i = g_p(x)\}_{i=1}^{M}$, the non-conformal prediction $\hat{y}$ and uncertainty $\hat{\sigma}$ are computed as the empirical mean and standard deviation of the samples, respectively. The probabilistic methods include Monte Carlo Dropout, Bayes by Backprop, and ensemble methods, which differ in how stochasticity is introduced into the model.

Deterministic methods

We implement two approaches that directly estimate both the SOC value and its uncertainty: quantile regression and a heteroscedastic Gaussian model.

Quantile regression[S1] is a method for directly estimating quantiles that is routinely used within conformal prediction frameworks.[S2,S3] Quantile regression utilizes the pinball loss function

$$\mathcal{L}_{\text{pinball}}(q, \hat{q}; \tau) = \tau \max(0, q - \hat{q}) + (1 - \tau) \max(0, \hat{q} - q), \tag{S1}$$

where τ is the desired quantile, q is the true value, and $\hat{q}$ is the predicted quantile. The flexibility of this loss function allows it to be applied to a variety of models such as CNNs [S2] and random forests.[S4]

In this work, we train a CNN to directly predict the 5th, 50th, and 95th percentiles of the SOC distribution. Here, we define the loss function as

$$\mathcal{L}_\tau(y, \hat{y}, \hat{\sigma}) = \mathcal{L}_{\text{pinball}}(y, \hat{y} + z\hat{\sigma}; 0.95) + \mathcal{L}_{\text{pinball}}(y, \hat{y} - z\hat{\sigma}; 0.05) + \mathcal{L}_{\text{pinball}}(y, \hat{y}; 0.5), \tag{S2}$$

where y is the true SOC value, $\hat{y}$ and $\hat{\sigma}$ are the predicted SOC value and standard deviation from the CNN, and $z \approx 1.64$ is the z-score for 90% coverage. Note that the loss function itself would allow the CNN to directly predict a 90th-percentile prediction interval, but this breaks consistency with the other heuristic methods.

Next, we consider a heteroscedastic Gaussian model as proposed by Nix and Weigend.[S5] More recently, this technique was used by Kendall and Gal[S6] to model aleatoric uncertainty in deep learning models. Here, we assume a Gaussian error model where the variance σ^2 is a function of the input x

$$p(y|x; \theta) = \mathcal{N}\left(\hat{y}(x), \hat{\sigma}(x)\right). \tag{S3}$$

The model $g_d(x)$ is trained by minimizing the negative log-likelihood that reduces to

$$\mathcal{L}(y, \hat{y}, \hat{\sigma}) = \frac{1}{2}\log\left(2\pi\hat{\sigma}^2\right) + \frac{(y-\hat{y})^2}{2\hat{\sigma}^2}. \tag{S4}$$

Probabilistic methods

A natural way to view probabilistic methods is through a Bayesian perspective. Here, the model is treated as a Bayesian neural network whose parameters are random variables $\theta \sim p(\theta)$. When given a training set $D = \{(x_i, y_i)\}_{i=1}^N$, the posterior distribution is defined by Bayes' theorem

$$p(\theta|D) = \frac{p(D|\theta)p(\theta)}{p(D)}. \tag{S5}$$

In general, computing the posterior distribution exactly is intractable because it requires evaluating the marginal likelihood

$$p(D) = \int p(D|\theta)p(\theta)d\theta, \tag{S6}$$

which involves integrating over all model parameters.

To address this, two major classes of approximate inference are commonly used: variational inference (VI) and Markov Chain Monte Carlo (MCMC). While MCMC offers strong theoretical guarantees and growing tooling support through libraries such as PyMC and Pyro,[S7,S8] VI remains the more popular choice in deep learning due to its compatibility with modern frameworks. Here, we focus on VI-based methods selected for their ease of adoption, computational efficiency, and accessible theoretical foundations.

Variational inference approximates the posterior distribution $p(\theta|D)$ using a simpler distribution $r(\theta;\phi)$ from a chosen family of distributions. The distribution's parameters ϕ are optimized to match the posterior distribution by minimizing the KL divergence between the two distributions $\mathrm{KL}(r_\phi(\theta)||p(\theta|D))$. This is equivalent to minimizing the negative evidence lower bound (ELBO)

$$\mathcal{L}(\phi) = \mathrm{KL}\left(r_\phi(\theta)||p(\theta)\right) - \mathbb{E}_{r_\phi(\theta)}\left[\log p(D|\theta)\right]. \tag{S7}$$

A method to apply variational inference to neural networks is using Bayes by Backprop.[S9] In this approach, the model weights θ are treated as random variables drawn from a Gaussian distribution $r(\theta;\phi) = \mathcal{N}(\mu_r, \sigma_r)$, parameterized by $\phi = [\mu_r, \sigma_r]$, with a prior distribution $p(\theta) = \mathcal{N}(\mu_p, \sigma_p)$. Under this parameterization, the loss function (S7) is reduced to

$$\mathcal{L}(\phi) = ||y - g_p(x;\theta)||^2 + \mathrm{KL}\left(r(\theta)||p(\theta)\right), \tag{S8}$$

where

$$\mathrm{KL}\left(r(\theta)||p(\theta)\right) = \frac{1}{2}\left(2\log\left(\frac{\sigma_p}{\sigma_r}\right) + \frac{\sigma_r^2 + (\mu_r - \mu_p)^2}{\sigma_p^2} - 1\right). \tag{S9}$$

Since the weights and output of the neural network are random variables, the gradients of the loss function must be computed via sampling. This is achieved through the reparameterization trick,[S10] where samples are generated as

$$\theta = \mu_r + \sigma_r \odot \epsilon, \tag{S10}$$

with $\epsilon \sim \mathcal{N}(0,1)$.

Monte Carlo (MC) Dropout[S11] is another method to approximate Bayesian inference in neural networks. Dropout[S12] was originally introduced as a regularization technique that randomly deactivates neurons during training by setting their outputs to zero with probability p_{drop}. Traditional dropout is disabled during inference.

MC Dropout departs from the traditional usage of dropout by keeping dropout active during inference. Gal and Ghahramani[S11] showed that a neural network with dropout can be interpreted as an approximation to a deep Gaussian process, a Bayesian model. MC Dropout effectively places Bernoulli distributions over the network weights, creating a simplified variational distribution. Binary masks that randomly "drop" connections during both training and inference can be viewed as samples from this distribution. Gal and Ghahramani demonstrated that training a neural network with dropout and L_2 regularization is equivalent to minimizing the negative evidence lower bound (ELBO) in variational inference, establishing dropout as a valid Bayesian approximation technique.

In practice, we perform multiple forward passes through the network with dropout enabled, each producing a different prediction. These predictions can be viewed as samples from an approximate posterior predictive distribution. By averaging these samples, we obtain an approximation of the Bayesian predictive mean, while their variance provides an estimate of the uncertainty.

Deep Ensembles[S13] represent another popular sampling-based method for estimating uncertainty based on bootstrapping. The technique involves training multiple neural networks with identical architectures but different random initializations and training data orderings. During inference, the prediction and uncertainty estimates are computed from the statistics of the ensemble predictions

$$\hat{y} = \frac{1}{M}\sum_{i=1}^{M} g(x;\theta_i), \quad \hat{\sigma}^2 = \frac{1}{M}\sum_{i=1}^{M}(g(x;\theta_i) - \hat{y})^2, \tag{S11}$$

where M is the number of ensemble members, $g(x;\theta_i)$ is the prediction of the ith ensemble member, and θ_i is the set of parameters for the ith ensemble member.

While Deep Ensembles were not originally formulated as a Bayesian method,[S13] recent research has established significant connections between ensemble techniques and Bayesian inference. Wilson and Izmailov[S14] argued that ensemble members effectively sample different modes of the posterior distribution, with each network converging to a distinct local optimum in the loss landscape. This multi-modal exploration provides a more diverse representation of plausible parameter values than single-mode approximations like MC Dropout. Further theoretical work by Wild et al.[S15] established rigorous links between Deep Ensembles and variational Bayesian methods, while Loaiza-Ganem et al.[S16] demonstrated that ensembles implicitly perform a form of empirical Bayes estimation. These connections help explain why Deep Ensembles often outperform explicit Bayesian approximations in practice, particularly for uncertainty estimation in complex data distributions and out-of-distribution detection.

CNN Architecture

The CNN contains multiple convolutional blocks followed by dense blocks. The deterministic methods (quantile regression and heteroscedastic Gaussian) share a similar architecture: 2 convolutional blocks with 128 filters each with a kernel size of 3, followed by 2 dense blocks with 128 neurons each and a dropout rate of 0.1. For probabilistic methods (MC Dropout,

Deep Ensembles, and Bayes by Backprop), we use 2 convolutional blocks with 64 filters each with kernel size of 3, followed by 3 dense blocks with 128 neurons each.

Method-specific implementations include:

- **MC Dropout**: Higher dropout rate (0.2) in dense layers and 30 forward passes with dropout active during inference to obtain uncertainty estimates.

- **Deep Ensembles**: Standard dropout rate (0.1) and consists of 10 independent models trained with different initializations using Huber loss with delta 1.0.

- **Bayes by Backprop**: Uses variational dense layers where weights are sampled from Gaussian distributions. The prior distribution has mean $\mu_p = 0$ and variance $\sigma_p^2 = 0.0025$. Variance is parameterized as $\sigma_p^2 = \log(1 + \exp(\rho_p))$ for numerical stability. Weight parameters μ_p are initialized uniformly in $[-1/\sqrt{d}, 1/\sqrt{d}]$ (where d is the input dimension) and ρ_p parameters are initialized from $\mathcal{N}(-3, 0.1)$. Inference uses 30 weight samples to generate predictions. When calculating the KL divergence within the loss function, the value is normalized by the number of weight parameters. No dropout is used in the dense blocks.

All models were trained using the Adam optimizer with a learning rate of 1e-4 and batch size of 32. Early stopping was applied based on the loss on the calibration set, with a maximum of 200 epochs and a patience of 20. A weight decay of 1e-4 was used for all models except for the Bayes by Backprop and heteroscedastic models, which used no weight decay. The Bayes by Backprop model used a maximum of 300 epochs and a patience of 40, while the heteroscedastic model used 400 epochs and a patience of 40, along with a linear warmup period of 30 epochs. Gradient clipping of 1.0 was applied to all models, except for MC dropout and Bayes by Backprop, which used no gradient clipping.

Conditional Coverage

In this section, we evaluate the conditional coverage of our framework by testing model performance across different data strata. This contrasts with marginal coverage which measures coverage across the entire dataset. While conformal prediction guarantees valid marginal coverage, it does not offer formal guarantees for conditional coverage. Nonetheless, conditional coverage more accurately reflects real-world performance, especially in subsets where model accuracy or uncertainty calibration may degrade. As such, it is a critical metric for assessing the practical reliability of our uncertainty estimates.

Conditional coverage analysis is inherently open-ended and flexible, as there are countless ways to stratify the data. In this work, we examine two straightforward stratification schemes, acknowledging that this is not exhaustive. First, we assess conditional coverage based on soil organic carbon levels, dividing the dataset into high-carbon ($> 3.5\%$) and low-carbon ($\leq 3.5\%$) samples. Second, we evaluate performance by geographical region, grouping samples by Alberta, Manitoba, Saskatchewan, Vancouver Island (British Columbia), Lower Mainland (British Columbia), and Midwestern United States.

Table S1 presents the performance metrics for high-carbon and low-carbon samples across all UQ methods. We observe no notable differences between methods within each group. However, the mean absolute error (MAE) is nearly twice as high for the high-carbon samples, suggesting greater difficulty in predicting SOC at elevated levels, possibly due to reduced spectral informativeness. Importantly, the magnitude of the error does not increase faster than the prediction values themselves; in fact, the mean absolute percentage error (MAPE) is lower for high-carbon samples than for low-carbon ones. These findings suggest that while absolute errors increase with SOC level, model calibration remains consistent, reinforcing the reliability of the uncertainty estimates across carbon strata.

Table S1: Prediction performance metrics for high-carbon and low-carbon samples across all UQ methods.

Model	High Carbon (>3.5%)			Low Carbon (≤3.5%)		
	MAE	MAPE	RMSE	MAE	MAPE	RMSE
Heteroscedastic Gaussian	0.742	0.146	0.919	0.450	0.306	0.631
Quantile Regression	0.712	0.137	0.897	0.423	0.301	0.576
Bayes By Backprop	0.666	0.131	0.843	0.412	0.268	0.588
MC Dropout	0.761	0.148	0.966	0.419	0.269	0.582
Deep Ensembles	0.650	0.125	0.816	0.443	0.291	0.611

Table S2 presents uncertainty metrics for high-carbon and low-carbon samples across all UQ methods after conformalization. Conditional coverage differs slightly between groups: high-carbon samples are mildly under-covered, while low-carbon samples are slightly over-covered. The difference in MAE between strata is reflected in the median predicted uncertainty, which is higher for the high-carbon samples. Notably, the correlation between absolute error and predicted uncertainty is very low for the high-carbon group, suggesting the model struggles to gauge its own error when spectral informativeness is reduced. Among the methods, the heteroscedastic Gaussian and deep ensemble models are least affected, maintaining a positive correlation with prediction error.

Table S2: Uncertainty metrics for high-carbon and low-carbon samples across all UQ methods after conformalization.

Model	High Carbon (>3.5%) / Low Carbon (≤3.5%)		
	Coverage (90%)	Median[$\hat{\sigma}$]	Corr[$\lvert\text{error}\rvert, \hat{\sigma}$]
Heteroscedastic Gaussian	0.862 / 0.904	0.825 / 0.549	0.086 / 0.474
Quantile Regression	0.854 / 0.929	0.806 / 0.571	-0.098 / 0.330
Bayes By Backprop	0.859 / 0.915	0.760 / 0.576	-0.158 / 0.336
MC Dropout	0.846 / 0.929	0.931 / 0.574	-0.112 / 0.414
Deep Ensemble	0.856 / 0.938	0.700 / 0.651	0.060 / 0.299

Table S3 summarizes the sample distribution by geographical region, and Table S4 reports the mean absolute error (MAE) by region and UQ method. Across regions, most UQ methods perform comparably, with a few notable exceptions: MC Dropout underperforms on Alberta samples but excels on Manitoba samples; the heteroscedastic Gaussian model shows elevated

error in Saskatchewan; and Bayes by Backprop achieves the lowest MAE on Vancouver Island. Regional MAE varies substantially, with the Midwest US exhibiting the lowest error and Manitoba the highest. These differences largely reflect disparities in sample size across regions.

Table S3: Geographical distribution and SOC statistics by region for the entire dataset.

Region	Samples	Mean	Std Dev	Min–Max Range
Alberta	127	5.374	1.197	0.690 – 6.800
Lower Mainland	104	4.445	1.127	1.500 – 6.830
Manitoba	44	3.009	0.878	0.880 – 4.880
Midwest US	374	1.533	0.822	0.280 – 5.710
Saskatchewan	119	2.667	1.044	0.480 – 6.160
Vancouver Island	133	5.314	1.052	2.840 – 6.950
**Total**	**901**	**3.190**	**1.916**	**0.280 – 6.950**

Table S4: Mean Absolute Error (MAE) by Region and UQ method.

Region	Het. Gaussian	Quantile Regression	Bayes by Backprop	MC Dropout	Deep Ensemble
Alberta	0.654	0.621	0.607	0.773	0.587
Lower Mainland	0.663	0.630	0.632	0.664	0.629
Manitoba	0.861	0.815	0.787	0.730	0.775
Midwest US	0.348	0.350	0.332	0.334	0.342
Saskatchewan	0.661	0.536	0.572	0.569	0.598
Vancouver Island	0.860	0.847	0.717	0.840	0.768
**Average**	0.674	0.633	0.608	0.652	0.616

Table S5 reports the conformalized 90% coverage by region and UQ method. Most methods achieve coverage close to the nominal 90% level across regions, with values ranging from 81.8% to 96.8%. While slight under-coverage is observed in most regions, the Midwest US stands out with consistent over-coverage.

Table S5: Conformalized 90% Coverage by Region and UQ method.

**Region**	**Het. Gaussian**	**Quantile Regression**	**Bayes by Backprop**	**MC Dropout**	**Deep Ensemble**
Alberta	0.890	0.874	0.913	0.835	0.898
Lower Mainland	0.875	0.875	0.856	0.894	0.875
Manitoba	0.818	0.750	0.795	0.795	0.818
Midwest US	0.936	0.957	0.941	0.952	0.968
Saskatchewan	0.832	0.908	0.866	0.882	0.891
Vancouver Island	0.827	0.812	0.820	0.835	0.797
**Average**	0.863	0.863	0.865	0.866	0.874

Overall, these results indicate that while some regional variation in performance and calibration exists, the conformal prediction framework provides stable and reliable uncertainty estimates across diverse environmental and geographical conditions.

References

(S1) Koenker, R.; Bassett Jr, G. Regression quantiles. *Econometrica: journal of the Econometric Society* **1978**, 33–50.

(S2) Romano, Y.; Patterson, E.; Candes, E. Conformalized quantile regression. *Advances in neural information processing systems* **2019**, *32*.

(S3) Angelopoulos, A. N.; Bates, S. A gentle introduction to conformal prediction and distribution-free uncertainty quantification. *arXiv preprint arXiv:2107.07511* **2021**,

(S4) Meinshausen, N.; Ridgeway, G. Quantile regression forests. *Journal of machine learning research* **2006**, *7*.

(S5) Nix, D. A.; Weigend, A. S. Estimating the mean and variance of the target probability distribution. Proceedings of 1994 ieee international conference on neural networks (ICNN'94). 1994; pp 55–60.

(S6) Kendall, A.; Gal, Y. What uncertainties do we need in bayesian deep learning for computer vision? *Advances in neural information processing systems* **2017**, *30*.

(S7) Salvatier, J.; Wiecki, T. V.; Fonnesbeck, C. Probabilistic programming in Python using PyMC3. *PeerJ Computer Science* **2016**, *2*, e55.

(S8) Bingham, E.; Chen, J. P.; Jankowiak, M.; Obermeyer, F.; Pradhan, N.; Karaletsos, T.; Singh, R.; Szerlip, P.; Horsfall, P.; Goodman, N. D. Pyro: Deep universal probabilistic programming. International Conference on Learning Representations (ICLR). 2019.

(S9) Blundell, C.; Cornebise, J.; Kavukcuoglu, K.; Wierstra, D. Weight uncertainty in neural network. International conference on machine learning. 2015; pp 1613–1622.

(S10) Kingma, D. P.; Welling, M. Auto-encoding variational bayes. 2013.

(S11) Gal, Y.; Ghahramani, Z. Dropout as a bayesian approximation: Representing model uncertainty in deep learning. international conference on machine learning. 2016; pp 1050–1059.

(S12) Srivastava, N.; Hinton, G.; Krizhevsky, A.; Sutskever, I.; Salakhutdinov, R. Dropout: a simple way to prevent neural networks from overfitting. *The journal of machine learning research* **2014**, *15*, 1929–1958.

(S13) Lakshminarayanan, B.; Pritzel, A.; Blundell, C. Simple and scalable predictive uncertainty estimation using deep ensembles. *Advances in neural information processing systems* **2017**, *30*.

(S14) Wilson, A. G.; Izmailov, P. Bayesian deep learning and a probabilistic perspective of generalization. *Advances in neural information processing systems* **2020**, *33*, 4697–4708.

(S15) Wild, V. D.; Ghalebikesabi, S.; Sejdinovic, D.; Knoblauch, J. A rigorous link between

deep ensembles and (variational) Bayesian methods. *Advances in Neural Information Processing Systems* **2023**, *36*, 39782–39811.

(S16) Loaiza-Ganem, G.; Villecroze, V.; Wang, Y. Deep Ensembles Secretly Perform Empirical Bayes. *arXiv preprint arXiv:2501.17917* **2025**,
